# Effect of high-fluoride dentifrice and bracket bonding composite material on enamel demineralization adjacent to orthodontic brackets *in vitro*

**DOI:** 10.4317/jced.57673

**Published:** 2021-05-01

**Authors:** Paulo Silva-Fialho, Robson Ferreira, José Leal, Cínthia Tabchoury, Gláuber Vale

**Affiliations:** 1 Restorative Dentistry Department, Federal University of Piauí, Teresina, Brazil; 2 Physiological Sciences Department, Piracicaba Dental School, Piracicaba, Brazil

## Abstract

**Background:**

There is a lack of information about the association of high-fluoride dentrifrice and fluoride-containing bonding material to prevent enamel white spot lesions development adjacent to brackets. The aim of this in vitro study was to evaluate the effect of high-fluoride dentifrice and fluoride-containing bonding material on enamel demineralization adjacent to orthodontic brackets.

**Material and Methods:**

Forty-eight enamel specimens with 7x7x2 mm were obtained from bovine incisors. Orthodontic brackets were bonded with fluoride-containing resin composite (OrthoCem®) or fluoride-free low viscosity resin. The specimens were submitted to an 8-day pH cycling that consisted in the daily immersion of specimens in the demineralizing solution for 4 h and in artificial saliva for 20 h in an incubator at 37° C. The treatments consisted in 5 min-immersion between the cycles of fluoride (F) suspensions containing 275 µg F/mL, 1,250 µg F/mL or distilled water (negative control). The 275 and 1,250 µg F/mL concentrations were used to simulate salivary dilution of 1,100 and 5,000 µg F/g dentifrices during toothbrushing. After the experiment, cross-sectional hardness was performed to analyze the lesion area of the specimens. Tukey post hoc test after two-way ANOVA with p at 5% was used as statistical analysis.

**Results:**

The specimens treated with high-fluoride dentifrice showed significantly less demineralization in comparison with the other treatments (*p*>0.05). There was a significant difference in the cross-sectional hardness values for the specimens bonded with OrthoCem when compared to the low viscosity resin (*p*>0,05).

**Conclusions:**

The use of high-fluoride dentifrice associated with fluoride-containing bonding material promoted a greater reduction of enamel demineralization adjacent to orthodontic brackets.

** Key words:**Demineralization, dentifrice, fluoride, bonding materials, orthodontic brackets.

## Introduction

White spot lesions are early manifestations of dental caries in enamel and it is frequently observed adjacent to the orthodontic brackets (1). These lesions are favored by orthodontic appliances that retain the biofilm combined with poor hygiene of the patients and long term of treatment (2). Indeed, orthodontics patients are in the high-risk group of dental caries development, particularly white spot lesions. Therefore, preventive measures are recommended to reduce the demineralization of dental tissues like dietary control, regular professional prophylaxis, use of F-varnish, and F-dentifrice to cite some (3). The use of fluoride-containing materials for bonding orthodontic brackets have also been investigated, but with weak evidence of effectiveness (4).

Fluoride mouth-rinse solutions are also routinely prescribed as a protocol for controlling dental caries in patients under orthodontic treatment by orthodontists (5). However, it has been observed that these prescriptions have not shown effectiveness since they are dependent on the patient’s collaboration, which does not usually occur because it consists in a modification in their routine of daily hygiene (6).On the other hand, the use of high-fluoride dentifrice (5,000 µg F/g) has been shown effective results on the reduction of enamel demineralization (7-9) and could be adopted without difficulty for the orthodontic patient as a biofilm control method, replacing conventional dentifrice (3). Also, this dentifrice provides higher fluoride release in biofilm (10-12) and saliva (13,14), which could act as F-reservoirs in the oral cavity.

Despite the promising results on enamel demineralization obtained with the use of high-fluoride dentifrice, there is a lack of studies in the literature to recommend it as a protocol for the management of white spot lesions in patients that are under orthodontic treatment. On the other hand, the use of fluoride-containing composite resins for bracket bonding showed a weak evidence in the reduction of enamel demineralization, needing more studies with robust design to subsidize their use (15,16). Therefore, the evaluation of the synergistic effect of F from the bonding material associated with the one from F-dentifrice on enamel demineralization is relevant, since there are few well-designed studies for this purpose. Thus, this *in vitro* study evaluated the effect of high-fluoride dentifrice associated to a bonding-brackets fluoride-containing resin composite on enamel demineralization adjacent to orthodontic brackets.

## Material and Methods

Forty-eight enamel specimens with dimensions 7x7x2 mm were acquired from bovine incisors. Those teeth were prior sterilized in 10% formaldehyde solution for 10 days (17) and then selected by surface hardness average using a microdurometer (Future Tech FM Hardness Tester, Future Tech Corporation, Kawasaki, Japan) accopled to a Knoop penetrator with a load of 25g for 5s (18). The overall average of the specimens was 346.2 (±32.56) Kg/mm2 and they were random-allocated to the different treatments (bonding composite materials and dentifrices, n=8 in each group). The sample size was determined based on previous findings using the same experimental protocol, with a statistical power higher than 0.8. After allocation in the groups, no statistical difference was observed in the surface hardness of the specimens (ANOVA, *p*>0.05), attesting homogeneity among the groups.

The surface of the specimens was protected with adhesive tape with a square window made with a 3 mm side. Then, it was conditioned with phosphoric acid (FGM, Joinville, Brazil) for 30 seconds, washed with distilled water, and dried with air jets. Metallic orthodontic brackets (Edgewise Slim – Slot 022 REF 10.65.203, Morelli, Nickel Free, São Paulo, Brazil) were placed and bonded on the window with fluoride-containing composite resin for bonding (OrthoCem®, FGM, Joinville, Brazil) or low viscosity resin fluoride-free (Natural Flow, Nova DFL, Taquara, Brazil) and photoactivated as recommended by the manufacturers. After that, the adhesive tape was removed, and the specimen’s surface was cleaned with gauze. The information about materials composition is displayed in Table 1.

**Table 1:**
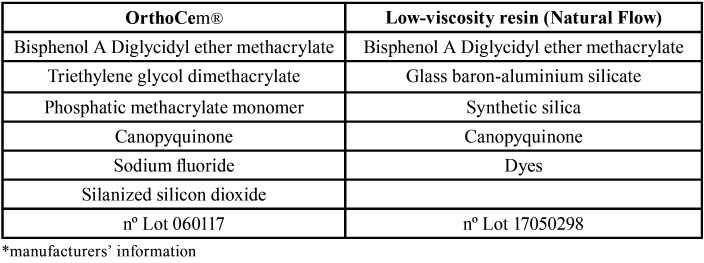
Composition of materials used for bracket bonding*.

The demineralizing solution was prepared to be 50% saturated regarding the enamel solubility with 0.05 M acetate buffer (pH 5.0). Thus, 0.05 mol/L acetate buffer, pH 5.0, containing 1.28 mmol/L Ca, 0.74 mmol/L *Pi*, and 0.03 μg F/mL was prepared from the salts of Ca(NO3)2.4H2O, KH2PO4, and NaF, respectively. This solution was used to simulate caries lesions in enamel specimens exposed to pH cycling. A solution of 1.5 mmol/L Ca, 0.9 mmol/L P, 150 mmol/L KCl, 0.05 mg F/mL in 0.1 mol/L Tris buffer, pH 7.0, was also prepared to simulate the components present in human saliva and served as a remineralization solution (19).

The specimens were divided randomly into pairs (one specimen with OrthoCem® and other with low viscosity resin) in three groups: suspensions with 1,250 µg F/mL or 275 µg F/mL and deionized and distilled water as the negative control. The 275 and 1,250 µg F/mL concentrations simulate the salivary dilution during a toothbrush with 1,100 and 5,000 µg F/g dentifrices, respectively.

The experiment was performed in 24-well plates containing 2 ml per well of the assigned solution (de or remineralization solutions and treatments) with the specimens fixed in holders to facilitate the immersion in the solutions. The specimens were then immersed in the treatments for 5 minutes. Then, they were washed with distilled water for 1 minute, immersed in the demineralizing solution and left for 4 h in an incubator at 37°C. Thereafter, a second wash with distilled water for 1 minute and immersion in treatment for 5 min was accomplished. After the treatments, the specimens were washed and kept individually in artificial saliva overnight in the incubator to simulate the remineralization process. The solutions were changed daily. After the eighth cycle, the specimens remained in artificial saliva for another 24 h.

**Figure 1:**
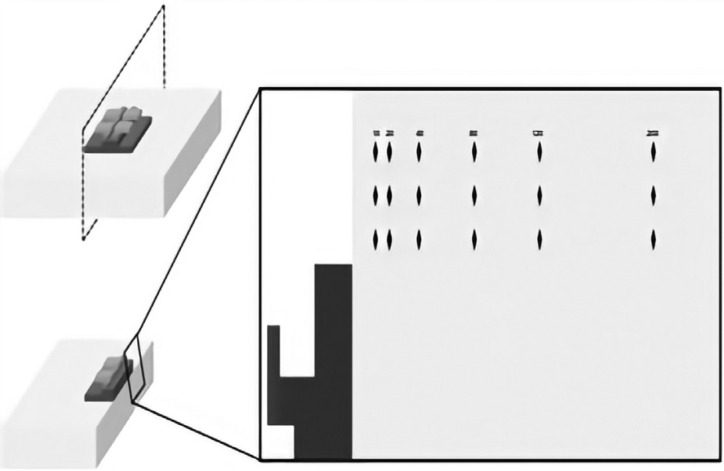
Scheme of enamel cross-sectional hardness analysis. After the enamel cross section, the indentations were made at diferrent distances from the surface.

**Figure 2:**
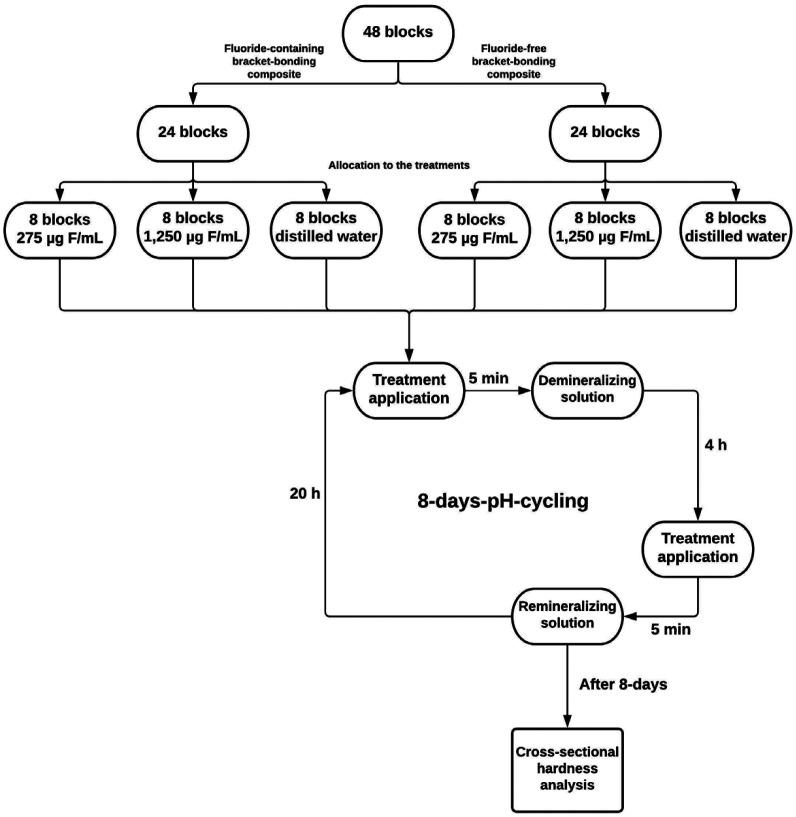
Flowchart of the experiment and sample distribution.

After the experiment, the enamel specimens were cross-sectioned with a diamond disc, adjacent to the bonded bracket (without removing it), embedded in acrylic resin and polished. Cross-sectional hardness was measured in three lines (one immediately adjacent to the bracket and the others 100 µm apart from the previous one) using distances of 10, 20, 40, 80, 120, and 200 μm from enamel surface with a microhardness tester (Future Tech FM Hardness Tester, Future Tech Corporation, Kawasaki, Japan) accopled a Knoop penetrator with a load of 25g for 5s (Fig. 1) (18). The hardness loss was used as an indicator of enamel demineralization. Also, the lesion area (ΔS) for each treatment was calculated by the subtraction of the area under the curve of the sound enamel from the area under the curve of the demineralized enamel (20). The flowchart (Fig. 2) summarizes step-by-step the methodology used.

For the statistical analyses, data were analyzed using SAS software (version 9.0) for Windows with *p* fixed at 5%. After verification of equality of variance and normal distribution of errors for the response variables, they were transformed into a base 10-logarithm. Two-way ANOVA was performed (considering the dentifrice and bonding resin composites as factors) followed by the Tukey post hoc test.

## Results

Two-way ANOVA showed a significant effect for the factors under study (dentifrices and bonding material, *p*<0.05), but not for the interaction (*p*>0.05) for the studied variables cross-sectional hardness and ΔS. Table 2 shows the cross-sectional hardness average in the enamel adjacent to orthodontic brackets bonded with OrthoCem® according to the distance from the surface, and it can be observed a higher hardness value in the specimens where the high-fluoride dentifrice was used, and it occurs more noticeable in the superficial layers (*p*<0.05). Although the treatment with 5,000 µg F/g dentifrice leads to higher hardness values, no statistical difference among treatments was observed from 40 µm-depth on (*p*>0.05).

**Table 2:**
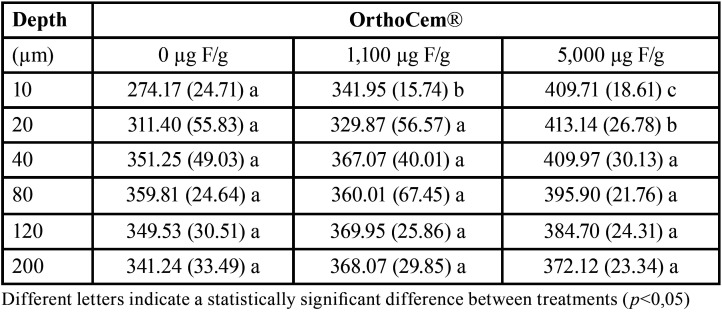
Mean (±SD) of cross-sectional hardness in enamel adjacent to orthodontic brackets bonded with fluoride-containing resin composite (OrthoCem®) according to the treatments (n=8).

Table 3 shows a cross-sectional hardness average in enamel adjacent to orthodontic brackets bonded with low viscosity resin. The treatment with 5,000 µg F/g dentifrice leads to higher hardness values in all depths evaluated (*p*<0.05).

**Table 3:**
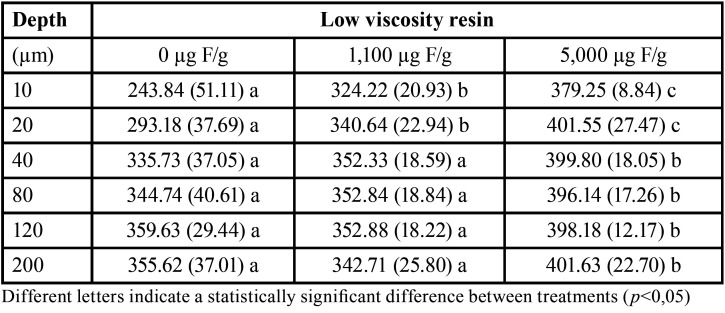
Mean (±SD) of cross-sectional hardness in enamel adjacent to orthodontic brackets bonded with low-viscosity resin according to the treatments (n=8).

**Figure 3:**
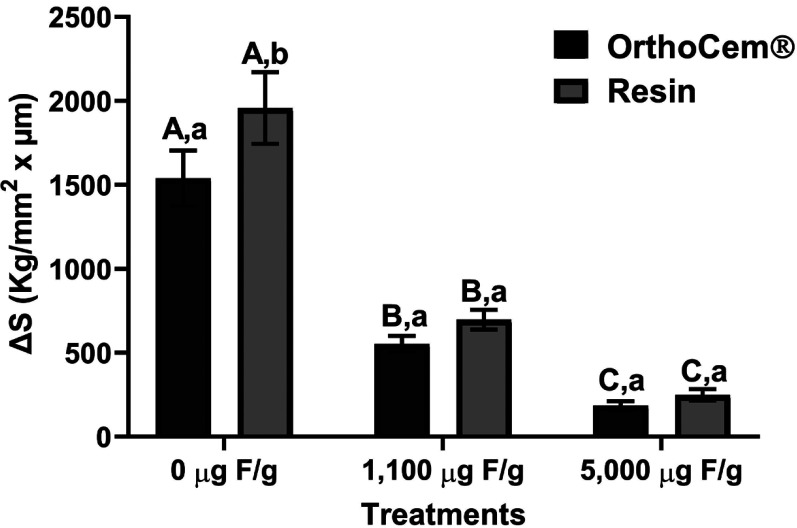
Mean (±SD) of the lesion area (ΔS) according to the treatments and bonding materials applied (n=8). Capital letters represent differences between dentifrices within each bonding material and lower case represent differences between bonding materials within each dentifrice (*p*<0.05).

Figure 3 shows the lesion area average in the specimens according to the treatments and bonding materials applied. The specimens treated with high-fluoride dentifrice presented a lower lesion area than the specimens treated with the other dentifrices irrespective of bonding material (*p*<0.05). No difference was observed between the bonding materials, except for placebo treatment, which specimens bonded with OrthoCem® had a lower lesion area than that bonded with low-viscosity resin (*p*<0.05).

## Discussion

Although the advances in orthodontic materials and treatment mechanics, the placement of fixed appliances is yet related to a high risk of white spot lesions development (21). Studies have demonstrated that caries lesions around orthodontic brackets could be reduced (21,22) or even totally inhibited with the use of F-dentifrice combined with a mouth-rinse (23). However, this treatment has low adequacy considering the absence of patient collaboration (6).

On the other hand, F-dentifrice is viewed as the most reasonable approach to manage tooth decay (24) and the dose-response between F-concentration in dentifrices and the preventive effect on enamel caries is well-elucidated (25). Particularly, the impact of high-fluoride products as dentifrices on the reduction of demineralization is already shown (8,25), and could be corroborated in the present study, since the high-fluoride dentifrice reduced the enamel demineralization compared the other treatments (Fig. 1).

Regarding the cross-sectional hardness analysis, it can be observed in  Table 1 and  Table 2 that the average in the enamel adjacent to orthodontic brackets increases proportionally in relation to the concentration of fluoride in the treatments, corroborating with other studies (7-9). This result confirms the effective reduction of enamel demineralization by fluoride but also demonstrates that its concentration in dentifrices is directly proportional to their caries-preventive effect (24,25). Also, the lesion area was lower for the specimens treated with high-fluoride dentifrice than the other treatments. These findings are in line with others *in vitro* (8) and in situ (7,9) studies.

Regarding the materials used for bonding procedures of orthodontic brackets to the enamel, it is possible to notice a difference in the cross-sectional hardness values between the specimens bonded with OrthoCem® or low viscosity resin, the first one having a lower demineralization. This difference could be ascribed to the fluoride release from the fluoride-containing resin composite. Despite the efficacy of this material observed in this study, the literature has indicated low effectiveness for fluoride-containing resins composite due to the short term of fluoride release compared to the longevity of orthodontic treatments (around 2 years duration) and the high risk of bias in the studies analyzed (3,4). This can be explained because of the greater complexity of the oral environment that cannot be totally simulated in laboratory studies.

The shear bond strength of the composites was not evaluated in this study since it was observed that it does not significantly influence the effect of reducing enamel demineralization of fluoride-releasing composites (26). However, a previous study has shown that OrthoCem® has a similar shear bond strength of Transbond XT, which is commonly used as a gold standard for orthodontic cement (27). Although there is a lack of comparison between the orthodontic cement and flowable resin used in this study in the literature, some studies using other flowable composite and orthodontic bonding systems have described a clinically satisfactory bond strength for both materials (28-30).

This study has limitations inherent to *in vitro* experiments, since dental demineralization occurred under controlled conditions in the laboratory, without the complexity present in oral cavity. However, the pH-cycling model used was responsive to the treatments applied, and lesion area has been used as indicator of mineral loss and gain (8). Besides, our findings are in accordance with what is reported in the literature regarding fluoride effect. Considering the heterogenicity of the results about bracket-bonding fluoride-containing composites, we believe that further clinical studies are necessary. However, we consider our study an early step in understanding the effect of high-fluoride dentifrice on the demineralization of enamel adjacent to orthodontic brackets and it may support further studies.

## Conclusions

We concluded that the association of high-fluoride dentifrice with a fluoride-containing resin composite for brackets bonding showed a synergistic effect, promoting a larger reduction of enamel demineralization adjacent to orthodontic brackets.
